# Advantage of proton-radiotherapy for pediatric patients and adolescents with Hodgkin’s disease

**DOI:** 10.1186/s13014-019-1360-7

**Published:** 2019-09-02

**Authors:** S. Lautenschlaeger, G. Iancu, V. Flatten, K. Baumann, M. Thiemer, C. Dumke, K. Zink, H. Hauswald, D. Vordermark, C. Mauz-Körholz, R. Engenhart-Cabillic, F. Eberle

**Affiliations:** 1 Klinik für Strahlentherapie und Radioonkologie, Klinikum der Philipps Universität Marburg, Baldingerstr, 35043 Marburg, Germany; 2 Technische Hochschule Mittelhessen, Institut für Medizinische Physik und Strahlenschutz, Gießen, Germany; 3 Klinik für Radio-Onkologie, Universitätsklinikum Heidelberg, Heidelberg, Germany; 4 Klinik und Poliklinik für Strahlentherapie, Universitätsklinikum der Martin-Luther-Universität Halle-Wittenberg, Halle, Germany; 5 Abteilung für Pädiatrische Hämatologie und Onkologie, Universitätsklinikum Gießen, Gießen, Germany; 6 Department für operative und konservative Kinder- und Jugendmedizin, Universitätsklinikum der Martin-Luther-Universität Halle-Wittenberg, Halle, Germany; 7 Marburg Ion-Beam Therapy Center (MIT), Marburg, Germany

## Abstract

**Abstract:**

Radiotherapy is frequently used in the therapy of lymphoma. Since lymphoma, for example Hodgkin’s disease, frequently affect rather young patients, the induction of secondary cancer or other long-term adverse effects after irradiation are important issues to deal with. Especially for mediastinal manifestations numerous organs and substructures at risk play a role. The heart, its coronary vessels and cardiac valves, the lungs, the thyroid and, for female patients, the breast tissue are only the most important organs at risk. In this study we investigated if proton-radiotherapy might reduce the dose delivered to the organs at risk and thus minimize the therapy-associated toxicity.

**Methods:**

In this work we compared the dose delivered to the heart, its coronary vessels and valves, the lungs, the thyroid gland and the breast tissue by different volumetric photon plans and a proton plan, all calculated for a dose of 28.8 Gy (EURO-NET-PHL-C2). Target Volumes have been defined by F18-FDG PET-positive areas, following a modified involved node approach. Data from ten young female patients with mediastinal lymphoma have been evaluated. Three different modern volumetric IMRT (VMAT) photon plans have been benchmarked against each other and against proton-irradiation concepts. For plan-evaluation conformity- and homogeneity-indices have been calculated as suggested in ICRU 83. The target volume coverage as well as the dose to important organs at risk as the heart with its substructures, the lungs, the breast tissue, the thyroid and the spinal cord were calculated and compared. For statistical evaluation mean doses to organs at risk were evaluated by non- parametric Kruskal-Wallis calculations with pairwise comparisons.

**Results:**

Proton-plans and three different volumetric photon-plans have been calculated. Proton irradiation results in significant lower doses delivered to organ at risk. The median doses and the mean doses could be decreased while PTV coverage is comparable. As well conformity as homogeneity are slightly better for proton plans. For several organs a risk reduction for secondary malignancies has been calculated using literature data as reference.

According to the used data derived from literature especially the secondary breast cancer risk, the secondary lung cancer risk and the risk for ischemic cardiac insults can be reduced significantly by using protons for radiotherapy of mediastinal lymphomas.

**Conclusion:**

Irradiation with protons for mediastinal Hodgkin-lymphoma results in significant lower doses for almost all organs at risk and is suitable to reduce long term side effects for pediatric and adolescent patients.

**Electronic supplementary material:**

The online version of this article (10.1186/s13014-019-1360-7) contains supplementary material, which is available to authorized users.

## Introduction

Hodgkin’s disease is interdisciplinary treated. It’s incidence follows a bimodal distribution regarding the age of onset [1]. One peak is reached at the age of 20 years and the other one around 70 years of age [1]. Nowadays, over all stages more than 80% of the patients can be cured on a long-term scale [1]. Therefore, evolving therapy strategies over the last decades aimed mainly at reduced therapy-associated toxicities. This resulted in balanced multidisciplinary concepts of deescalated chemotherapeutic approaches and shrinking volumes and minimized doses in radiotherapy. Depending on stage radiotherapy concepts evolved from extended field/mantle field [2–5] to involved field [6–12] and finally for certain stages to involved node [13]. At the same time the dose was deescalated from 40 Gy [2–4] to 30 Gy [6–12] and finally down to 20 Gy [14–16], also depending on stage and chemotherapeutics applied. While carefully adjusting and balancing the aim of progressions free survival versus critical toxicities from chemotherapy or radiotherapy [15–21], study protocols for minors and adolescents followed a similar approach. When treating affected lymphatic tissues in the mediastinal region different sensitive organs at risk are vulnerable to both chemotherapeutics and radiation. Major late toxicities that came up decades after initial treatment and that are related to initial radiotherapy are cardiovascular events with a focus at ischemic [22, 23] and valvular diseases [23]. Secondary malignancies, mainly breast-cancer [24–26], lung cancers [25, 27], soft-tissue sarcomas [28–31] and thyroid-cancers [32, 33] also increase in incidence years or decades after the treatment of Morbus Hodgkin. Most negative effects exhibit a dose dependency for both, chemotherapeutic agents and radiotherapy. Additionally, they depend on the irradiated volume of the organ at risk. Thus, lower doses and lower irradiated volumes lead consequently to lower toxicities [22–27, 29, 32–34].

Despite the already ongoing reductions of radiotherapy-dose and irradiated volumes modern radiotherapy with highly conformal treatment techniques, such as image-guided intensity-modulated radiation therapy (IMRT) and proton therapy (PT) might further reduce the dose delivered to organs at risk and in consequence diminish the toxicity of radiotherapy [35]. However, IMRT-approaches feature one drawback at this point. Due to the many different gantry-angles they distribute low doses of radiation over large fractions of the body with possible consequences for late onset secondary malignancies. That is a problem which can in general be avoided by using intensity modulated proton therapy (IMPT). Especially proton therapy (PT) for mediastinal target volumes is supposed to reduce the dose to critical organs at risk such as the heart, the lungs and breasts. Therefore, reducing the risk of cardiac morbidity and second malignancies [28, 36–38]. However, the steep dose fall-off in IMPT leaves little room for error and caution is needed when using this technique to avoid increased risk of recurrences [39]. Techniques for motion management (i.e. 4D-CT, internal target volume concepts, breath-hold techniques) and robustness optimized IMPT plans are therefore required. In the adult population particle therapy has been used in limited studies [40–43] but only few data [44] are available analyzing PT in pediatric or adolescent patients suffering from Hodgkin’s disease.

In this study we report on our dosimetric comparison of different IMRT techniques and PT in 10 underaged or young adult patients with mediastinal and paracardial Hodgkin’s disease. Our aim was to determine which technique best optimizes radiation dose distribution by minimizing dose to organs at risk with focus on the breast tissue, the lungs and the heart with its cardiac substructures while maintaining optimal coverage of the target volume.

### Methods and patients

We included ten different minor or young adult patients with Hodgkin’s disease in our study. At least mediastinal (m), in some cases also supraclavicular (sc), cervical (c) or axillary level III (a) affections were present. Two patients had solely upper mediastinal involvement, three only lower mediastinal disease and five patients feature combined upper and lower mediastinal involvement.

**Fig. 1:**
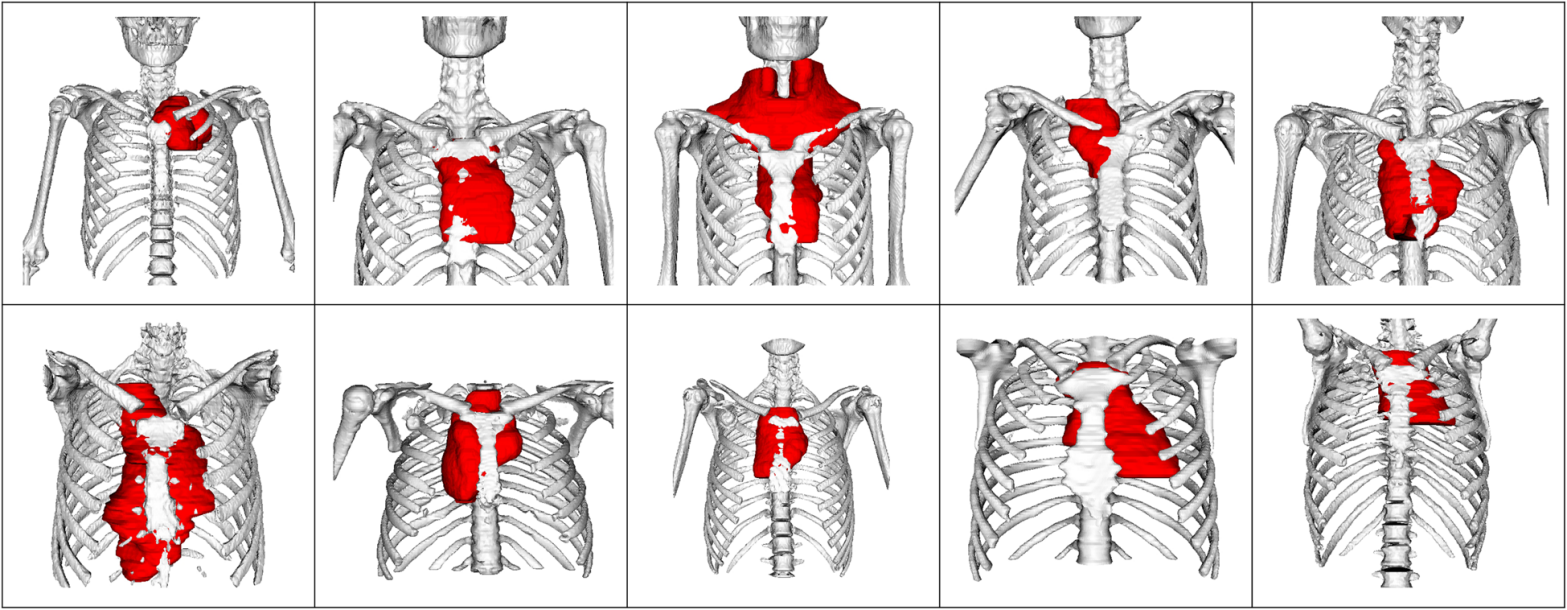
Target Volumes (PTV) for each of the ten included patients. Besides a mediastinal involvement some target volumes encompass axillary level III, supraclavicular or cervical lymphatic tissues as well

Figure 1 displays the PTV target volumes for all ten patients. Radiotherapy doses have been taken from the protocol of the pediatric and adolescent Euronet-PHL-C2 [20, 45] study. For the COPDAC-28 treatment arm a PTV dose of 28.8 Gy, 95% isodose encompassing the PTV, was used as dose prescription [45]. Target volume delineation was done according to study protocol. Thus, for GTV delineation PET positive residual areas after initial chemotherapy cycles have been used. Only partly PET positive tissues were encompassed in whole. For CTV definition 5 mm were added to delineated GTVs. PTV expansion was done by adding again 5 mm to the CTV.

Organs at risk have been delineated by experienced radiation oncologists and include the heart, the coronary vessels, the cardiac valves, the esophagus, the trachea, the lungs, the breast tissue, the spinal cord and the thyroid.

**Fig. 2:**
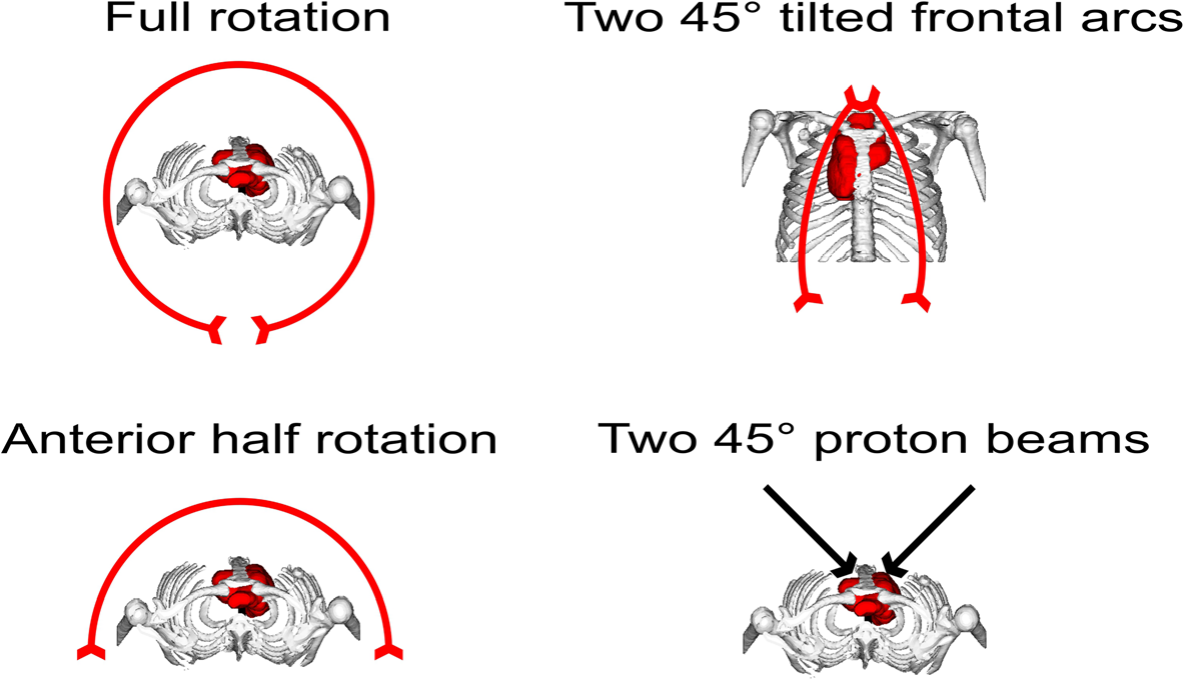
Schematics of the calculated proton- and IMRT plans. Full rotation, anterior half rotation and two non-coplanar 45° tilted quarter rotations have been benchmarked against the proton-plans

For each individual patient one Proton-Plan and three additional IMRT Plans with different Couch and Gantry Starting positions have been calculated. Intensity modulated Proton (IMPT) plans were calculated with 45° beams from each side of the patient. One axial full rotation IMRT, one axial half rotation IMRT and one IMRT plan with a 90° turned couch and 45° tilted 90° Arcs have been prepared (See Fig. 2 for additional explanation of the used techniques). The two 45° tilted arcs were based on the butterfly approach [46, 47] which was modified to better fit our clinical setup. All plan optimizations have been done using Varian Eclipse V13.7. Photon plans were calculated for a True-Beam 2.5 linear accelerator, proton- plans were used using the machine data of the Marburg Ion Therapy (MIT) center. The MIT facility has three 90° beamlines and one 45° beamline (IEC 61217). The lymphoma PTV can encompass besides the mediastinal region also supraclavicular and cervical ones. The 45° beamline was chosen in order to achieve short path lengths and avoid passing through critical organs like the lung. It also has been shown that the peculiarity of the lung tissue introduces an intrinsic dose modulation [48], which scales with the length of the tissue, imposing further optimization parameters which can be avoid. The movement mitigation technique we use is breath-hold with iso-layered rescanning [49]. 4D- CT- scans have also been available. Multi-field robust optimization on CTV was employed with Varian Eclipse V13.7 considering ±2 mm setup uncertainty and ± 3.5% range uncertainties. Generalized equivalent uniform dose (gEUD) values were 30.1 ± 0.3 respectively 28.8 ± 0.29. Plans have each been individually been optimized with first priority on PTV coverage (95% isodose covering the PTV). Secondary objectives were to minimize the heart-dose, the dose to cardiac-valves and the dose to coronary vessels, the minimal V_20Gy_ of the Lungs and V_4Gy_ of the breast tissue. Further organs at risk with importance include the esophagus, the spinal cord and, whenever PTV made it necessary, the thyroid. For the calculation of the body dose the body segment between the fourth cerebral and the first lumbar vertebrae has been utilized.

Mean DVHs involving all individual DVHs for each organ at risk were computed using Eclipse 13.7 (Varian) and 3DSlicer V4.8.1 [50]. In addition, mean doses for all organs at risk have been calculated and compared. Homogeneity- and conformity indices (HI and CI) were assessed according to ICRU83 [51] (see Eq.1 and Eq.2) For statistical evaluation non-parametric ANOVA with pair-wise comparisons (Kruskal-Wallis) of the mean-dose to organs at risk has been undertaken. A statistics software (SPSS V21.0, IBM, New York, USA) was utilized. Significance level was set at *p* < 0.05.

1 $$ {H}_I=\frac{D_{2\%}-{D}_{98\%}}{D_{50\%}} $$

Eq 1: Homogeneity Index [51] as defined in ICRU83

2 $$ {C}_I=\frac{V_{95}}{V_{PTV}} $$

Eq 2: Conformity Index [51] as defined in ICRU83.

## Results

**Fig. 3:**
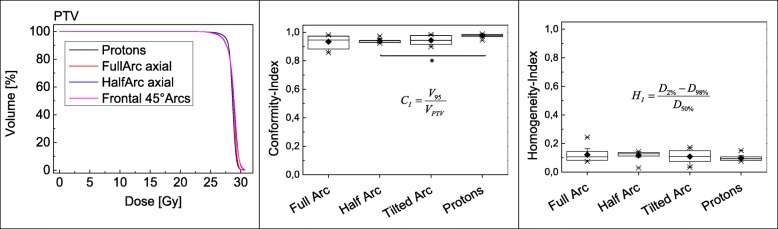
PTV-DVH, PTV-homogeneity- and -conformity-indices calculated according ICRU83 [51]

All IMRT plans as well as the IMPT plans feature excellent PTV coverage, high conformity and homogeneity for all patients. Figure 3 shows the averaged PTV-DVH. The averaged homogeneity- and conformity- indices for the PTV coverage were calculated according to ICRU83 [51]. Nevertheless, the proton plan is slightly superior especially regarding the conformity of the PTV coverage (Conformity- Index mean / median for proton-RT 0.97 and 0.98, for full arc- RT 0.93 and 0.95, for half arc-RT 0.94 and 0.94 and for tilted quarter arc-RT 0.94 and 0.94). Statistical significance with a *p*-value of *p* = 0.016 was reached for the comparison of the conformity between the anterior-half-rotation and the proton-plan. The comparison of protons versus full rotation IMRT missed significance by a hair’s breadth with a *p* value of 0.051. Other differences were not statistically significant.

**Fig. 4:**
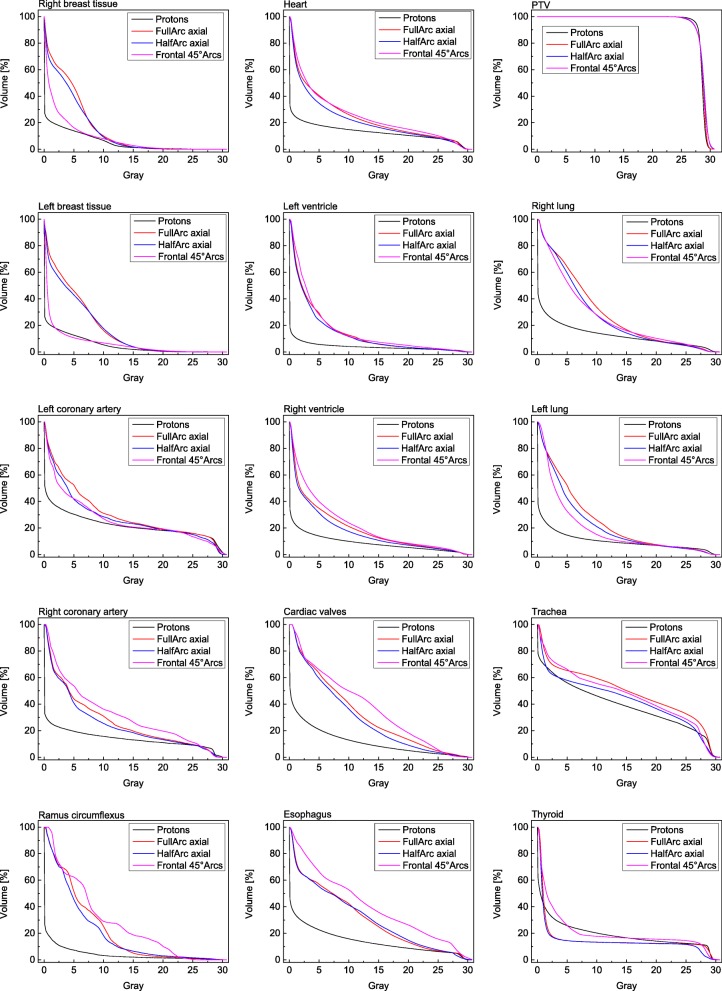
Mean-DVHs for the PTV and the organs at risk for the different planning-modalities. The protons feature, except for the thyroid, a superior sparing of organs at risk while PTV coverage of all plans is comparable

**Fig. 5:**
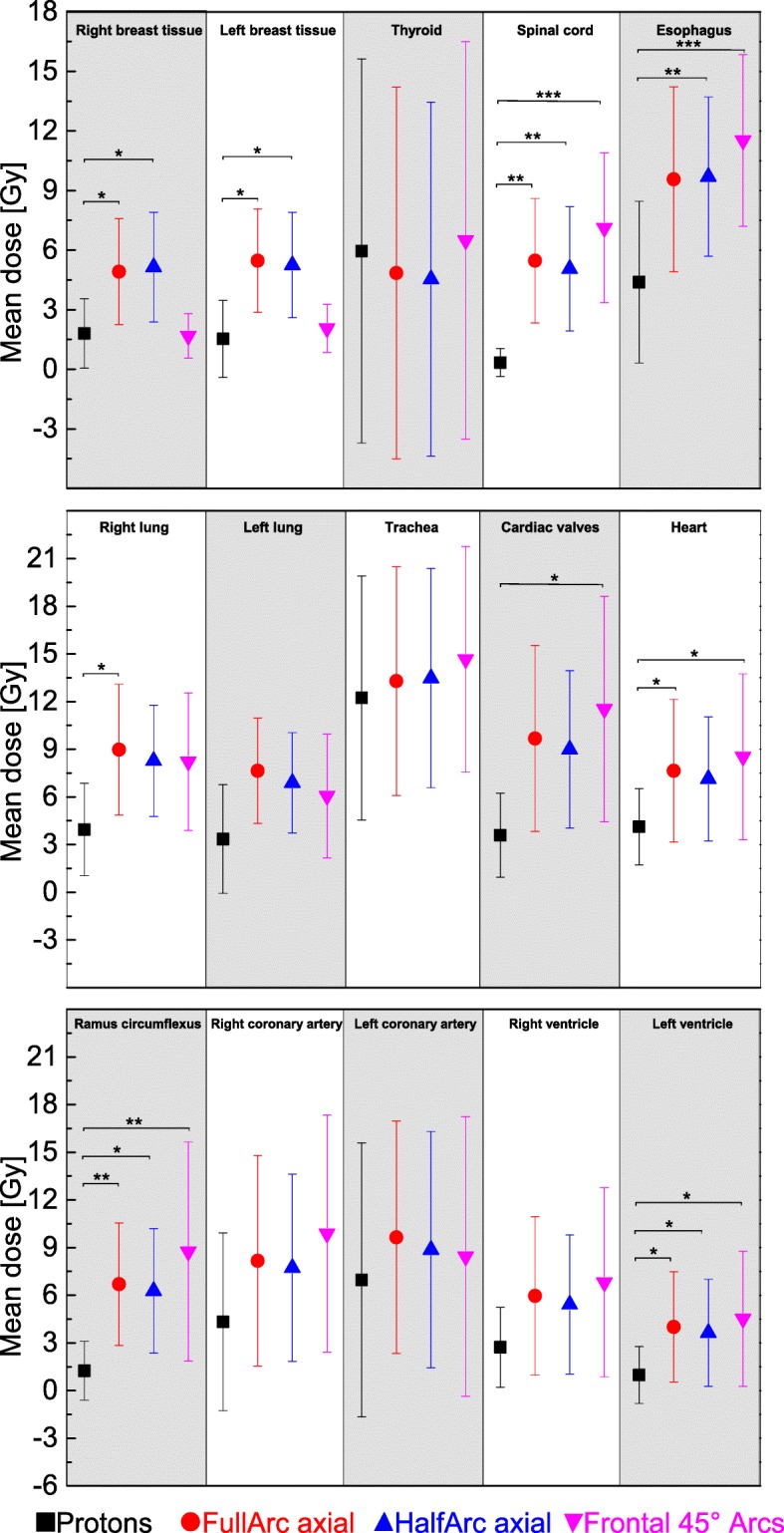
Mean dose and standard error for different photons or proton plans and for all delineated organs at risk. *P* values were calculated for mean dose, comparing proton and photon planning approaches. P values were given if significance has been reached. (*➔*p* = < 0.05; **➔*p* < 0.01; ***➔*p* < 0.001)

The mean-DVHs that were calculated for the heart, the cardiac valves, the left coronary artery, the right coronary artery, the ramus circumflexus, the left and right lung, the thyroid, the left and right breast tissue, the trachea and the esophagus can be seen in Fig. 4. Over the complete dose range the proton plans feature superior dose-sparing of organs at risk, especially in the lower and intermediate dose region of the DVHs. The mean dose delivered to the breast tissue is reduced by 3.1 Gy and 3.2 Gy comparing IMPT with full-arc or anterior half-arc IMRT at significance levels of *p* = 0.0026 and *p* = 0.05. Regarding the breast tissue the non-coplanar IMRT plans achieve a dose-sparing comparable to the proton-plans. Thus, in consequence, the mean dose to the heart is raised by 4.4 Gy (*p* = 0.012) to 8.5 Gy. Full and half in plane Arcs lead to mean heart doses around 3 Gy above the doses of the proton plans (*p* = 0.028 for full-arc compared to protons). Other cardiac substructures that have significantly been spared by using protons were the left ventricle (2.7Gy – 3.5 Gy mean dose reduction compared to the arc-rotations, *p*-values of 0.018, 0.035 and 0.012) and the ramus circumflexus (dose reductions of 5.0 Gy - 7.5 Gy, p-values 0.009, 0.024 and 0.01). Additionally, statistically significant mean-dose reductions were achieved for the esophagus and the spinal cord (Fig. 5 and Additional file 1: Table S1). The dose-sparing of anatomical features in the vicinity of the PTV is more limited compared to that of organs in the more extended neighborhood. That is valid for example for the left coronary artery, the right ventricle and also for the thyroid when the PTV extends to the cervical region (see Figs. 4 and 5). However, despite not all calculated mean doses to organs at risk reached statistical significance mean doses delivered by proton planes to the lungs, the right and left coronary arteries and the cardiac valves were lower compared to all three calculated photon plan varieties (Fig. 5 and Additional file 1: Table S1).

**Fig. 6:**
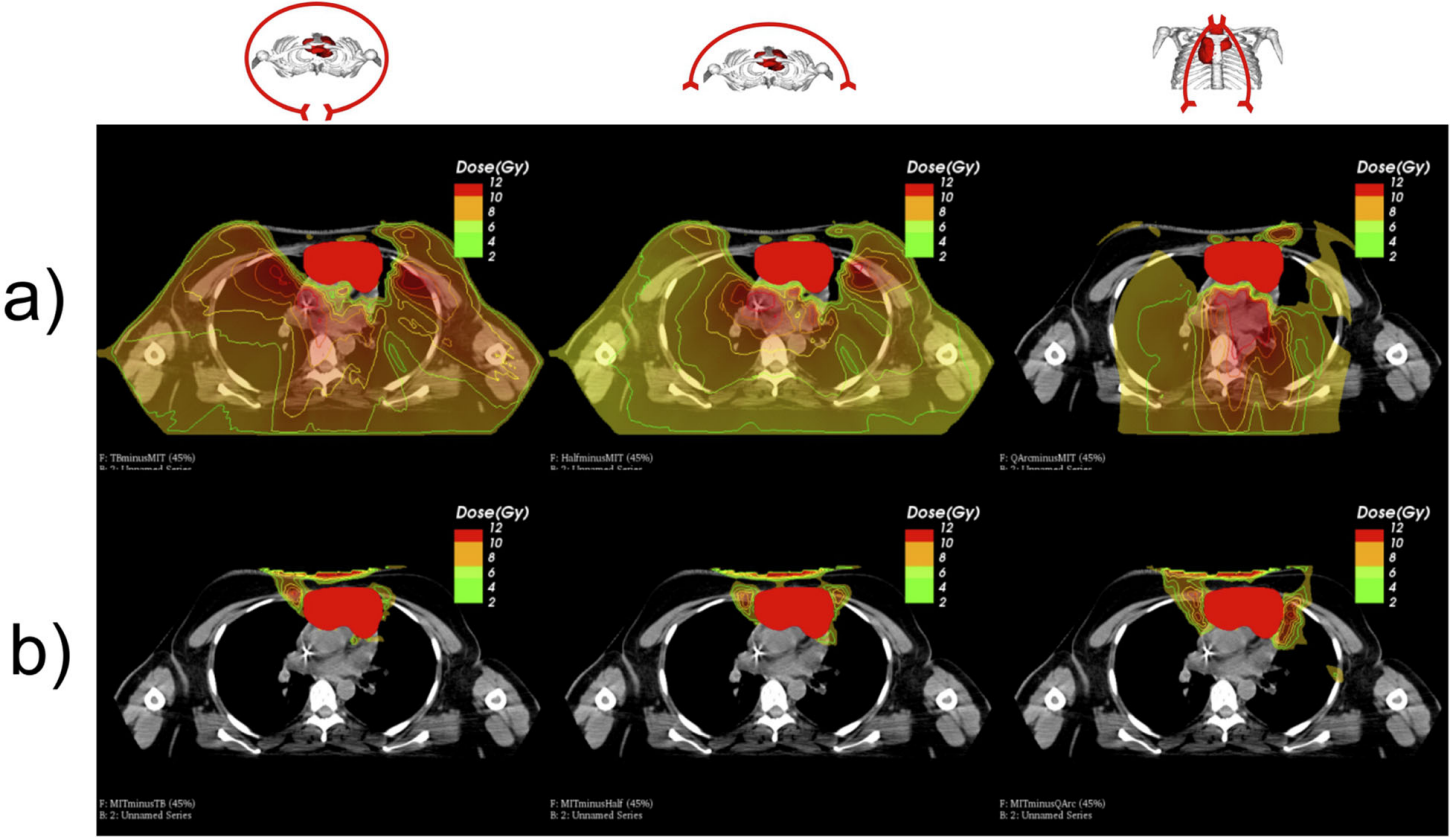
**a** Areas in which the VMAT / IMRT plans will deliver more dose to organs at risk or the body compartment. **b** Areas in which the proton plan delivers more dose to organs at risk or the body compartment compared to the VMAT / IMRT plant

To visualize the findings mentioned above, as an example, we plotted the dose difference between each photon and the proton plan for one patient for each plan. Figure 6 displays the results, an axial CT slice in the mediastinum has been chosen. The PTV has been delineated as filled red structure. Isodose-lines were plotted in addition to the colorwash dose representation. All IMRT plans distribute more dose to all organs at risk across the body. For the non- coplanar 45° quarter-rotations, as it has been already seen in the DVHs, while sparing dose to the breast tissue, the heart dose increases. Generally, in beam direction anterior to the PTV the proton plan delivers a higher dose to normal tissue. All relevant organs at risk receive lower doses with proton therapy.

**Fig. 7:**
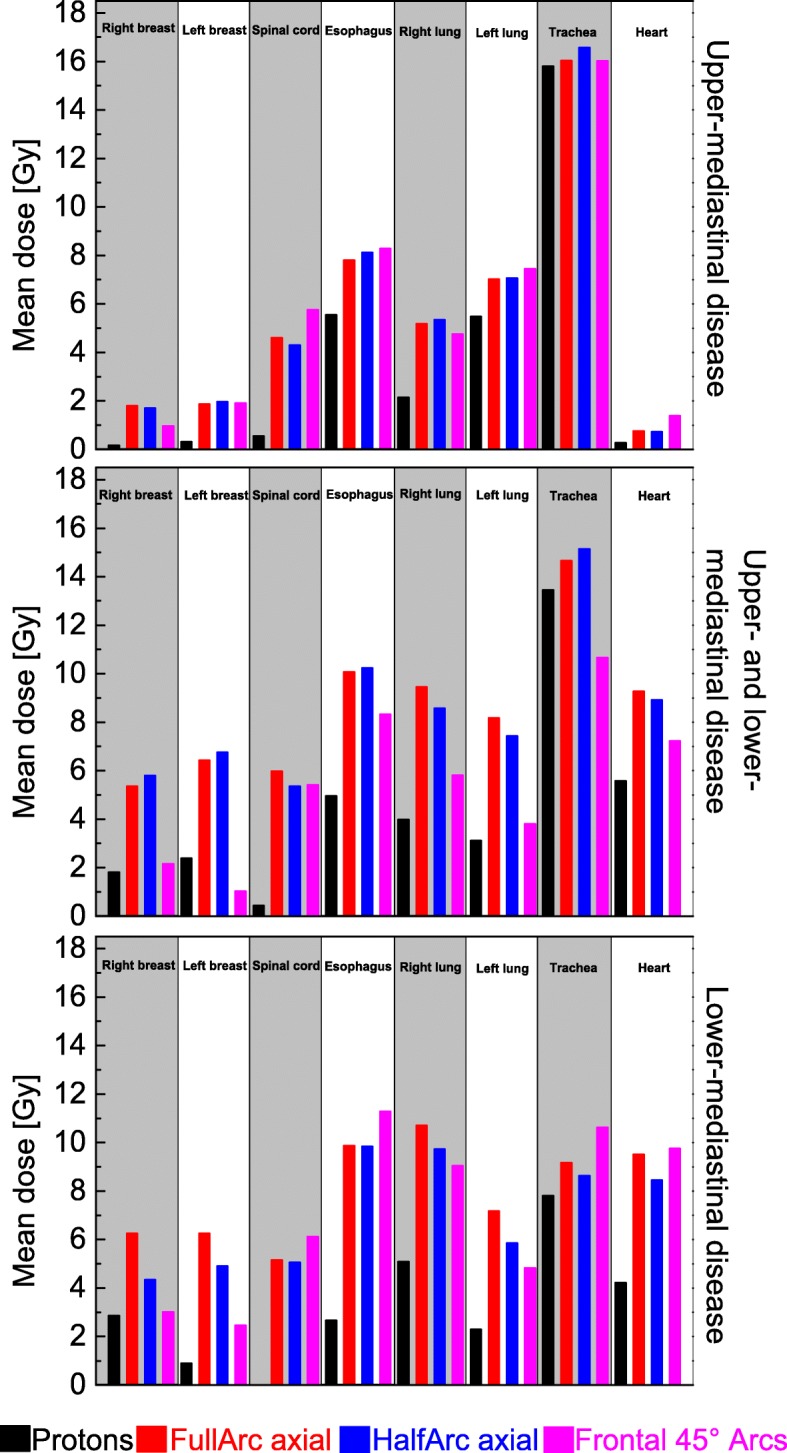
Comparison of the mean dose to organs at risk for upper, lower or combined upper and lower mediastinal radiotherapy treatment. Five patients had combined upper and lower mediastinal disease, 3 patients only lower and 2 only upper mediastinal disease

Subgroup analysis for upper, lower and combined mediastinal involvement has been undertaken. Upper mediastinum has been defined as the region between the thoracic inlet and the thoracic plane. Lower mediastinum was defined as the region below the superior mediastinum, with the diaphragm as caudal border. Two patients had solely upper mediastinal disease, three patients lower mediastinal lymphoma and five patients featured combined upper and lower mediastinal disease. As organs at risk for this subgroup analysis the breasts, the heart, the lungs, the trachea, the esophagus and the spinal cord have been selected. Figure 7 shows the mean doses to the organs at risk for upper, lower and upper and lower mediastinal disease (numeric values can be found in Additional file 1: Table S2).

The doses to organs at risk differ for the different mediastinal locations of Hodgkins disease. Upper mediastinal irradiation leads to higher doses to the trachea and the esophagus for all four different irradiation concepts. The lymphatic tissues in this region are in the direct vincinity of the two organs at risk so dose sparing with proton-radiotherapy is limited. For upper mediastinal involvement the heart and the breast tissue as organs at risk don’t play that role as for lower mediastinal disease. Nevertheless, proton-RT deliveres lower mean doses (right breast: 0.17 Gy, left breast: 0.32 Gy, heart: 0.27) compared to photon approaches (right breast: 0.97–1.8 Gy, left breast: 1.87–1.98 Gy, heart: 0.77–1.39 Gy).

Radiotherapy for lower or combined lower and upper disease lead to increased doses to the heart and the breast tissue for all techniques. While photon irradiation delivers 7.23–9.76 Gy to the heart and 2.47–6.76 Gy to the breasts, protons cut the dose to these two organs at risk to values below 2.9 Gy for the breast tissue and below 5.6 Gy for the heart.

Doses to the esophagus and the trachea are lower for all techniques compared to upper mediastinal disease. However, the dose reduction is pronounced for proton-RT.

**Fig. 8:**
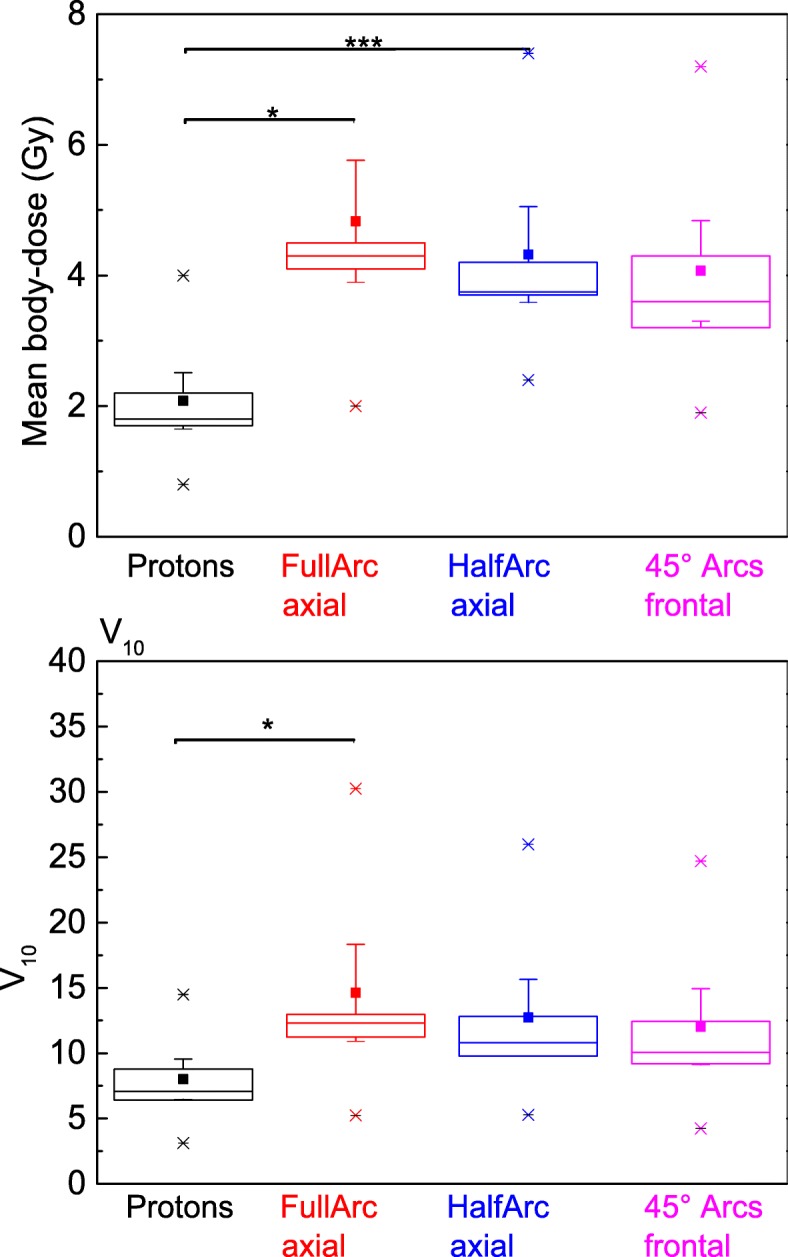
V10 and mean body dose for the body compartment between the fourth cerebral and the first lumbar vertebrae. As well median (line) as mean (square) doses are lower by the factor of 2–3 when comparing photon-RT-techniques with the proton-RT-technique. (Statistics: Kruskal-Wallis ANOVA with pairwise comparisons. Significance *p* = 0.025 for difference in V10 protons versus Full-ARC- RT, *p* = 0.017 for mean-dose protons versus FullArc-RT and *p* < 0.001 for protons versus Half-ARC-RT)

The dose delivered to the body, in our case the trunk between the fourth cerebral and the first lumbar vertebrae, has been calculated for all different four radiotherapy techniques. The V10 (Volume of the body receiving 10 Gy or more) and the mean- dose to the body are displayed in Fig. 8. Proton radiotherapy reduces the mean dose to the body roughly about the factor of 2 (1,8 Gy for proton-RT versus 4,3 Gy for full arc, 3.75 Gy for half-arc or 3,6 Gy for the tilted quarter arcs). The volume of the body-compartment receiving 10 Gy or more is reduced by about 30 - 50% when irradiating with protons (V10 = 7.1% for protons versus 12.3% for full arc, 10,8% for half arc and 10.1% for quarter arcs).

## Discussion

Therapy associated toxicities are major drawbacks in modern cancer treatment especially for the treatment of children and adolescents. For Hodgkin’s disease over the last decade radiotherapy as well as chemotherapy evolved to risk adapted concepts with, for radiotherapy, decreasing irradiated volumes and radiation doses. So far photons have been the preferred type of radiation whenever the necessity of radiotherapy arose. Recently modern volumetric intensity modulated techniques pushed back the conventional 3D approach and feature excellent conformity and homogeneity for PTV coverage. In the clinical routine, the IMRT is used in different forms. The IMRT techniques differ in terms of conformity and low dose exposure to organs at risk. Multiple arc techniques (butterfly VMAT) are described as a good option to achieve the most balanced compromise between high conformity and low dose exposure of organs at risk [47]. Further optimization of these modern IMRT techniques (e.g. full-arc butterfly VMAT) may lead to a risk reduction for cardiovascular events [46]. Nevertheless, one flaw of photons that cannot be resolved because of basic physics is the infinite range of photons. Whenever a photon beam enters the body every structure that lies in its way to the opposing body surface is going to receive some extend of radiation dose. That’s where particle beams, in our case protons, are in advantage. Due to their finite range and Bragg-peak energy deposition characteristics they feature both excellent homogeneity, conformity and a reduced dose delivered to organs at risk. Plan comparison studies are an effective instrument to show superiority of one radiotherapy plan over another. Though, one flaw of this method is that the general quality of the different plans has to be equal. Therefore, in our study we compared three different IMRT approaches with axial full-rotations, axial half-rotations or non-coplanar 45° rotations and protons plans with two fixed 45° beams. They all feature their unique characteristic of dose distribution to organs at risk. So far to our best knowledge no other study benchmarked different VMAT concepts against proton plans and almost all other planning studies so far used virtual proton facilities while we used the data of the real Marburg-Ion-beam Therapy-Center synchrotron. Also, we investigated underaged and adolescent patients or young adults under the age of 30 which has not systematically been done in the past.

For mediastinal location of Hodgkin’s disease important therapy related adverse effects are cardio-vascular events [22, 52–55], secondary cancers and hypothyroidism [56, 57]. Secondary cancers partly promoted by radiotherapy include for mediastinal tumors mainly breast cancer [26, 58–62], lung cancer [27, 63, 64], sarcoma [28–30] and thyroid cancers [65]. For all these cancers a radiation- dose and in the case of breast cancer also an irradiated area dependency has been derived over the decades in literature.

Of undisputed importance is of course the development of breast cancers in female Hodgkins’s disease survivors. The lifetime risk for females to develop breast cancer in Germany is roughly 13% [1]. Therefore, changes in relative risk might afflict many patients. Especially underaged and young adults under the age of 30 years have a significantly elevated risk of developing breast cancer within their lifespan. Hancock et al. [59] reported a strong dependency of the excess relative risk of developing breast cancer on the age of irradiation. Patients treated below the age of 20 suffer a 26 times elevated relative lifetime breast-cancer risk. For young adults treated for Hodgkin’s disease between 20 and 30 years of age the elevated relative risk was 7.8. A dependency on radiation dose for development of breast cancer has also been discussed. Travis et al. [26] demonstrated an elevated breast cancer risk with increasing fraction of the breast tissue receiving 4 Gy or more. Inskip et al. [58] found a linear slope of 0.27 Gy^− 1^ mean dose to the breast tissues for the increase in relative cancer risk. The findings were confirmed by several other studies [58, 60–62], however one has to mention that available data analyze cases mainly from the 70s and 80s when modern treatment techniques in radiotherapy were not available and applied doses have been retrospectively reconstructed using the available data (mainly 2-D radiographs). Nevertheless, with the data available we estimated the lifetime excess relative risks for developing breast cancer for each used technique. Table 1 shows the calculated values, the increase in relative risk has been assessed by multiplying the mean dose to the breast tissues with the data provided by Inskip et al. [58]. Clearly the two axial VMAT plans, the half- and the full rotation with mean doses to the breasts of around 5 Gy increases relative risks of patients under 30 years for lifetime breast cancer by up to 148%, while the proton and the non-coplanar VMAT treatment perform head-to-head, deliver mean doses of 1.5 to 2 Gy (relative risk for breast cancer increased by around 50% for both modalities). Both approaches offer excellent sparing of the breast tissue also by minimizing the breast volume receiving 4 Gy and above. For the non-coplanar tilted arc VMAT plan the sparing of the breast tissue leads to increased doses to other organs at risk. Particularly the heart receives an increased mean dose of 8.5 Gy while the axial VMAT plans deliver 7–7.5 Gy to the heart. The proton mean dose is 4.1 Gy. Nimwegen et al. reported an elevated relative risk for coronary heart disease of 7.4% per Gy mean heart dose [52]. We calculated our excess relative risk in Table 1 according to these data by multiplying the mean heart dose for each technique with 7.4% Gy^− 1^. The excess risk for heart diseases is reduced by 50% when using protons instead of photons. Interesting is that they also state that for patients below the age of 27 years the excess relative risk per Gray is even as high as 20%. That might even be of bigger importance for our pediatric and adolescent patients all aged below 30 years. For the cardiac substructures it has not been finally clarified if delineating and sparing the coronary arteries or the cardiac valves bears the possibility of reducing ischemic events or valvular disease. Retrospective data from large cohort analysis [22, 53, 54, 66] found either no significant dependency on dose to the coronary vessels and adverse effects or concluded that there might be some dependency but the suggested statistical model performed better by using the mean heart dose as independent factor. The mean dose delivered to the heart so far is the only validated predictor for radiation induced ischemic events. The impact of irradiation of the cardiac valves so far is not clear. Historical data from patients irradiated from the 70s to the 90s show an increase in valvular disease for Hodgkin survivors with high RT doses [55]. However, the administered doses to the valves were quite high and in the range between 20 Gy and 40 Gy or even above. Thus, despite the excellent dose reduction from 9 to 11.5Gy for the photon approaches to 3.6 Gy mean dose for the proton plan (see Fig. 5 and Additional file 1: Table S1), the clinical significance remains unclear. Finally, when dealing with cardiovascular toxicity one has to mention the excess risk due to anthracycline-containing chemotherapeutical regimes [22, 67, 68] when treating morbus Hodgkin. Long-term survivors of Hodgkin’s disease have an up to three-fold elevated risk of developing cardiac events when treated with anthracyclines solely [66]. Effects of chemotherapy and radiotherapy are additive [22, 66, 67] and up to now anthracyclines are an important part of all therapeutic regimes [5, 7, 8, 11–14, 18, 20, 21, 45, 69] used. As of more importance for long-term outcome, particularly for pediatric patients, a reduced cardiac toxicity from radiotherapy will be.

**Table 1:**
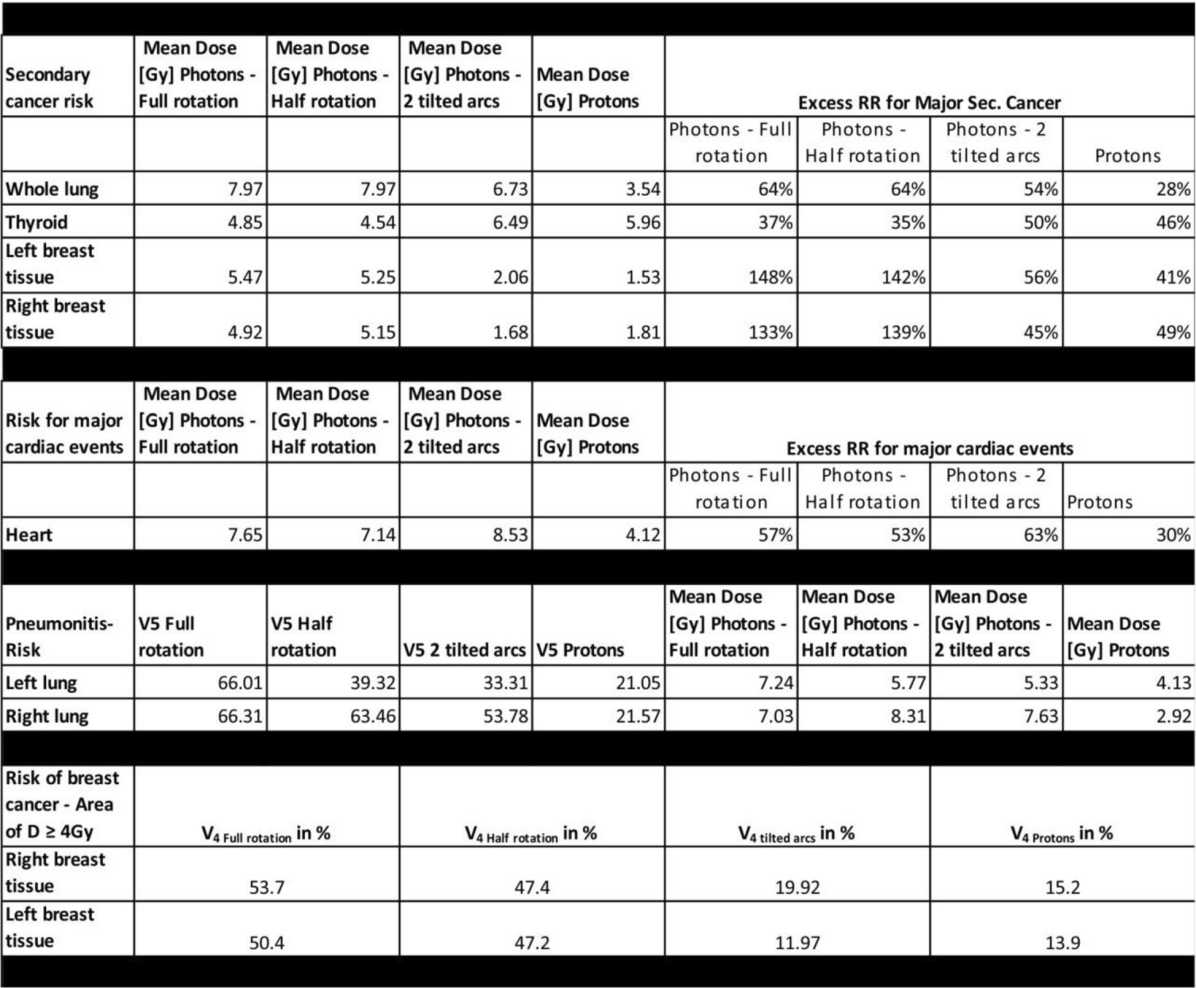
Estimation of excess risk for long term adverse effects for organs at risk. The lungs, the breast tissue, the heard and the thyroid were evaluated

Only few reports [27, 63, 70] investigated secondary lung cancers after treatment of Hodgkin’s or Non-Hodgkin’s disease. Travis et al. [27] report that mean lung doses of 5 Gy lead to a higher relative risk of developing lung cancer at the irradiated site up to decades after Hodgkin treatment but no quantitative risk assessment has been done. However, the effect is more pronounced for higher mean doses and does not exist for mean doses below 5 Gy. The risk estimate in Table 1 has been calculated according to the data published by Inskip et al. [64] for the development of lung- cancer after radiotherapy for breast cancer. Inskip et al. reported an increased relative risk of 8% Gy^− 1^ mean lung dose for secondary lung cancers [64]. We multiplied the mean dose to the whole lung with this value to estimate the elevated relative risk for secondary lung cancers. Due to the fact that the mean and median age of the patients in the study of Inskip et al. cannot be retrieved from the publication our calculations are only a rough estimate and can well underestimate the lung-cancer risk. But even with these data it can be clearly seen that the mean doses to the lungs are lower and the low-dose bath from VMAT is absent for proton therapy. Thus, this will not only decrease the risk for lung cancer but also the pneumonitis risk from irradiation. Abou-Yehia et al. [71] found an elevated pneumonitis risk if the mean lung dose exceeded 13.5 Gy or with increasing lung volume that received 5 Gy or above. Proton irradiation minimize both factors down to threefold smaller values. This should lead to a reduced rate of post-radiotherapy pneumonitis.

The induction of sarcoma by radiotherapy in survivors of childhood cancers, and especially in survivors of Hodgkin’s disease is important as well. Literature reports sarcoma to be the second most common malignancy in childhood cancer survivors [29, 30]. Radiotherapy increases the relative lifetime risk for secondary sarcoma depending on the RT-dose and the irradiated area [28, 30]. In contrast to the organ specific secondary malignancies like breast- or lung cancer, sarcoma can briefly occur in any location. There is no defined organ at risk except the irradiated body. Data from childhood survivor studies reported by Henderson et al. [29] suggest a dependency for the development of sarcoma from the applied dose per volume. They found elevated risks for areas irradiated with more than 10 Gy (Odds-Ratio for the development of sarcoma = 15.6 [29]). The higher dose regimes (> 30Gy) with even more elevated ORs are not reached in our study. We calculated in addition to the mean body dose the V_10_ which is the fraction of the body receiving a RT dose of 10 Gy or more. Using proton- RT as well the mean body dose as the V10 body volume is reduced by roughly a factor of two. Therefore, the induction of secondary sarcoma using proton-RT instead of photon-approaches might be reduced as well (see Fig. 8).

The thyroid has to be taken into account. Hypothyroidism with its onset approximately 5 years after the Hodgkin treatment seems to be multifactorial. Chemotherapy and Radiotherapy play their role as well. Due to the fact that again most data rely on old mantle-field or extended field treatments [56, 57] a risk- quantification up to now seems difficult. Undisputed should be that avoiding dose to the thyroid should be one goal when irradiating cervical lymphatic nodes. Unfortunately whenever the lymphatic nodes in the cervical region have to be irradiated the thyroid can, due to its adjacent position to the lymphatic structures, not be spared. Doses to the thyroid are especially increased for the techniques that use anterior fields only, in our case for the proton and the non-coplanar tilted 45° arcs. However, the other investigated beam- configurations deliver high doses as well. Despite the uncertainties for the hypothyroidism for the induction of secondary thyroid cancers a risk estimation was made by using the data published by Ron et al. [65]. They supposed an increased relative risk for thyroid cancers of 7.7% Gy^− 1^ mean dose to the organ at risk. Thyroid mean doses from our study have been multiplied with this value to assess the increase in relative risk for thyroid cancers. As to be expected both ventral only techniques feature a slightly increased relative risk for thyroid cancer of roughly 10–15% higher in relative risk compared to the axial VMAT techniques. Lifetime-risk for Thyroid cancer in Germany is 0.4% for men and 0.8% for women [1], slight increases of relative risks might therefore play not a major role. However literature hints that especially thyroid glands of children are vulnerable to radiation [65]. When irradiating target volumes in the upper mediastinum or cervical region even more attention is necessary to choose the optimal radiotherapy- technique.

Under discussion in literature is if patients with lower mediastinal disease benefit more than patients with upper mediastinal disease. 2018 the ILROG published a guideline [72] in which they proposed advantages especially for proton RT for lower mediastinal involvement. Recently Everett et al. [42] and Ntentas et al. [43] published data dealing with that issue as well. In both studies doses to organs at risk, in particular to the heart, the lungs and the breast tissue is lower for proton RT compared to IMRT photon approaches in lower mediastinal disease. The dose-sparing effect is pronounced for lower mediastinal disease, however as well existent for upper mediastinal involvement. In addition Ntentas at al [43]. confirmed a dosimetric advantage for proton-RT, regarding the organs at risk, for axillary involvement. In the study conducted by Everett et al. [42] deep inspiration breath hold for proton-RT featured no or only minimal dosimetric advantages compared to free breathing techniques when irradiating lower mediastinal lymphoma. Thus, breathhold-techniques might be dispensable which would simplify the proton-RT process. Comparing the two studies with our own results, the dose reduction comparing proton-RT with IMRT photon techniques is around the same scale we find in our study. Of course, numeric values are not one-to-one comparable due to different target volumes and anatomical features in each investigated case. For our cases investigated here, we also suppose that lower mediastinal disease benefits most from proton-RT in regard to the sparing of the heart, the lungs and the breast tissue. However, the usage of protons for upper mediastinal disease likewise spares that organs at risk to a limited extent. Thus, for patients under 30 we would recommend the usage of proton-RT for upper and lower localizations of mediastinal Hodgkin’s lymphoma.

## Conclusion

Proton therapy for mediastinal lymphoma reduces significantly the dose to organs at risk and the integral body dose. It might lead to reduced late toxicities and secondary malignancies. This is especially important for children and young adults. It should be considered for both sexes, as both male and female patients benefit from the unique features of particle irradiation. Whenever proton for mediastinal lymphoma is not available or technical not feasible the alternative photon concepts have to be chosen carefully. Depending on the used technique certain organs at risk, i.e. the breasts in young females, can be spared with higher priority. However, with all photon techniques that comes at the cost of higher doses to the other organs at risk. If available, proton therapy should be the standard pattern of care for mediastinal lymphoma for young adults below 30 years of age, no matter if male or female.

## Additional file

Additional file 1: **Table S1.** Mean dose and standard error for different photons or proton plans and for all delineated organs at risk. *P* values were calculated for mean dose, comparing proton and photon planning approaches. *P* value were given if significance has been reached. **Table S2.** RT doses to organs at risk, divided into upper-, lower- and combined mediastinal disease. (DOCX 35 kb)

## Data Availability

The datasets generated and the data analyzed are not publicly available.
