# Supplementary material for: Advantage of proton-radiotherapy for pediatric patients and adolescents with Hodgkin’s disease
Source: Radiat Oncol. 2019 Sep 2;14:157. doi: 10.1186/s13014-019-1360-7 (PMC6721251; doi:10.1186/s13014-019-1360-7)
Supplement: Supplementary file 1 — Table S1. Mean dose and standard error for different photons or proton plans and for all delineated organs at risk. P values were calculated for mean dose, comparing proton and photon planning approaches. P value were given if significance has been reached. Table S2. RT doses to organs at risk, divided into upper-, lower- and combined mediastinal disease. (DOCX 35 kb) [file 13014_2019_1360_MOESM1_ESM.docx]

Additional file

|  |  | Mean Dose [Gy] | Standard Deviation |  |
| --- | --- | --- | --- | --- |
| Right breast tissue | Protons | 1.81 | 1.75 | *p= 0.026 for Protons vs. Full-Arc  p= 0.05 for Protons vs. Half-Arc* |
|  | Full-Arc | 4.92 | 2.67 |  |
|  | Half-Arc | 5.15 | 2.76 |  |
|  | Tilted Quarter-Arcs | 1.68 | 1.12 |  |
| Left breast tissue | Protons | 1.53 | 1.94 | *p= 0.05 for Protons vs. Full-Arc  p= 0.008 for Protons vs. Half-Arc* |
|  | Full-Arc | 5.47 | 2.60 |  |
|  | Half-Arc | 5.25 | 2.65 |  |
|  | Tilted Quarter-Arcs | 2.06 | 1.21 |  |
| Thyroid | Protons | 5.96 | 9.67 |  |
|  | Full-Arc | 4.85 | 9.36 |  |
|  | Half-Arc | 4.54 | 8.91 |  |
|  | Tilted Quarter-Arcs | 6.49 | 10.01 |  |
| Spinal cord | Protons | 0.34 | 0.70 | *p= 0.001 for Protons vs. Full-Arc  p= 0.007 for Protons vs. Half-Arc p<0.001 for Protons vs. Quarter-Arcs* |
|  | Full-Arc | 5.47 | 3.13 |  |
|  | Half-Arc | 5.06 | 3.13 |  |
|  | Tilted Quarter-Arcs | 7.12 | 3.77 |  |
| Esophagus | Protons | 4.39 | 4.07 | *p= 0.047 for Protons vs. Half-Arc p= 0.006 for Protons vs. Quarter-Arcs* |
|  | Full-Arc | 9.57 | 4.66 |  |
|  | Half-Arc | 9.70 | 4.01 |  |
|  | Tilted Quarter-Arcs | 11.52 | 4.32 |  |
| Right lung | Protons | 3.95 | 2.91 | *p= 0.04 for Protons vs. Full-Arc* |
|  | Full-Arc | 8.98 | 4.12 |  |
|  | Half-Arc | 8.28 | 3.50 |  |
|  | Tilted Quarter-Arcs | 8.22 | 4.33 |  |
| Left lung | Protons | 3.35 | 3.42 |  |
|  | Full-Arc | 7.65 | 3.31 |  |
|  | Half-Arc | 6.89 | 3.16 |  |
|  | Tilted Quarter-Arcs | 6.06 | 3.90 |  |
| Trachea | Protons | 12.23 | 7.68 |  |
|  | Full-Arc | 13.29 | 7.20 |  |
|  | Half-Arc | 13.48 | 6.90 |  |
|  | Tilted Quarter-Arcs | 14.66 | 7.10 |  |
| Cardiac valves | Protons | 3.59 | 2.64 | *p= 0.028 for Protons vs. Quarter-Arcs* |
|  | Full-Arc | 9.68 | 5.86 |  |
|  | Half-Arc | 8.99 | 4.95 |  |
|  | Tilted Quarter-Arcs | 11.53 | 7.09 |  |
| Heart | Protons | 4.12 | 2.40 | *p= 0.028 for Protons vs. Full-Arc p= 0.012 for Protons vs. Quarter-Arcs* |
|  | Full-Arc | 7.65 | 4.49 |  |
|  | Half-Arc | 7.14 | 3.91 |  |
|  | Tilted Quarter-Arcs | 8.53 | 5.22 |  |
| Ramus circumflexus | Protons | 1.25 | 1.85 | *p= 0.009 for Protons vs. Full-Arc  p= 0.024 for Protons vs. Half-Arc p= 0.01 for Protons vs. Quarter-Arcs* |
|  | Full-Arc | 6.70 | 3.85 |  |
|  | Half-Arc | 6.28 | 3.92 |  |
|  | Tilted Quarter-Arcs | 8.75 | 6.89 |  |
| Right coronary artery | Protons | 4.33 | 5.60 |  |
|  | Full-Arc | 8.17 | 6.62 |  |
|  | Half-Arc | 7.73 | 5.89 |  |
|  | Tilted Quarter-Arcs | 9.88 | 7.46 |  |
| Left coronary artery | Protons | 6.97 | 8.62 |  |
|  | Full-Arc | 9.65 | 7.31 |  |
|  | Half-Arc | 8.87 | 7.44 |  |
|  | Tilted Quarter-Arcs | 8.44 | 8.79 |  |
| Right ventricle | Protons | 2.73 | 2.52 |  |
|  | Full-Arc | 5.96 | 4.99 |  |
|  | Half-Arc | 5.42 | 4.38 |  |
|  | Tilted Quarter-Arcs | 6.81 | 5.95 |  |
| Left ventricle | Protons | 0.98 | 1.79 | *p= 0.018 for Protons vs. Full-Arc  p= 0.035 for Protons vs. Half-Arc p= 0.012 for Protons vs. Quarter-Arcs* |
|  | Full-Arc | 4.01 | 3.47 |  |
|  | Half-Arc | 3.64 | 3.37 |  |
|  | Tilted Quarter-Arcs | 4.52 | 4.26 |  |

Table S1: Mean dose and standard error for different photons or proton plans and for all delineated organs at risk. P values were calculated for mean dose, comparing proton and photon planning approaches. P value were given if significance has been reached.

|  |  | Upper mediastinum | Lower mediastinum | Upper + lower mediastinum |
| --- | --- | --- | --- | --- |
|  |  | Mean dose [Gy] | Mean dose [Gy] | Mean dose [Gy] |
| Right breast tissue | Protons | 0.17 | 2.87 | 1.82 |
|  | Full-Arc | 1.81 | 6.26 | 5.36 |
|  | Half-Arc | 1.71 | 4.35 | 5.81 |
|  | Tilted Quarter-Arcs | 0.97 | 3.02 | 2.17 |
| Left breast tissue | Protons | 0.32 | 0.91 | 2.39 |
|  | Full-Arc | 1.87 | 6.26 | 6.43 |
|  | Half-Arc | 1.98 | 4.90 | 6.76 |
|  | Tilted Quarter-Arcs | 1.91 | 2.47 | 1.05 |
| Spinal cord | Protons | 0.56 | 0.02 | 0.44 |
|  | Full-Arc | 4.62 | 5.16 | 5.99 |
|  | Half-Arc | 4.30 | 5.06 | 5.36 |
|  | Tilted Quarter-Arcs | 5.76 | 6.13 | 5.41 |
| Esophagus | Protons | 5.55 | 2.67 | 4.96 |
|  | Full-Arc | 7.81 | 9.88 | 10.08 |
|  | Half-Arc | 8.13 | 9.85 | 10.24 |
|  | Tilted Quarter-Arcs | 8.30 | 11.30 | 8.34 |
| Right lung | Protons | 2.15 | 5.09 | 3.99 |
|  | Full-Arc | 5.19 | 10.72 | 9.46 |
|  | Half-Arc | 5.36 | 9.74 | 8.57 |
|  | Tilted Quarter-Arcs | 4.76 | 9.05 | 5.82 |
| Left lung | Protons | 5.49 | 2.30 | 3.12 |
|  | Full-Arc | 7.03 | 7.18 | 8.19 |
|  | Half-Arc | 7.07 | 5.86 | 7.44 |
|  | Tilted Quarter-Arcs | 7.45 | 4.84 | 3.82 |
| Trachea | Protons | 15.80 | 7.81 | 13.45 |
|  | Full-Arc | 16.04 | 9.17 | 14.66 |
|  | Half-Arc | 16.57 | 8.63 | 15.15 |
|  | Tilted Quarter-Arcs | 16.02 | 10.63 | 10.67 |
| Heart | Protons | 0.27 | 4.23 | 5.58 |
|  | Full-Arc | 0.77 | 9.52 | 9.27 |
|  | Half-Arc | 0.73 | 8.45 | 8.92 |
|  | Tilted Quarter-Arcs | 1.39 | 9.76 | 7.23 |

Table S2: RT doses to organs at risk, divided into upper-, lower- and combined mediastinal disease.
